# Case 5/2015 – 88-Year-Old Female with Chronic Coronary Artery Disease, Upper Limb Thrombosis, Atrial Fibrillation and Cardiac Arrest

**DOI:** 10.5935/abc.20150143

**Published:** 2015-11

**Authors:** Magaly Marçula, Vera Demarchi Aiello

**Affiliations:** Instituto do Coração (InCor) do Hospital das Clínicas da Faculdade de Medicina da Universidade de São Paulo, São Paulo, SP – Brazil

**Keywords:** Coronary Artery Disease, Upper Extremity Deep Vein Thrombosis, Atrial Fibrillation, Heart Arrest, Aged, In-stent Stenosis

An 88-year-old female was admitted due to pain and swelling of the left upper limb swelling (September 28, 2011).

She was first admitted to Instituto do Coração (InCor) at the age of 73 years (December 12, 1996), because of chest angina on great exertion for 8 months.

On that occasion, she reported arterial hypertension, glucose intolerance, hypercholesterolemia, hypertriglyceridemia, family history of sudden death, and smoking cessation at the age of 51 years.

Her physical examination evidenced heart rate of 60 bpm, blood pressure of 150/80 mmHg. Her heart, lung and abdomen examinations were normal. Her lower limbs showed no edema and her pulses were symmetrical.

The electrocardiogram (December 9, 1996) revealed sinus rhythm, an electrically inactive area in the inferodorsal wall, and ventricular repolarization changes with inverted T waves from V_1_ to V_5_ (Figure 1).

The laboratory tests (January 13, 1997) evidenced: glycemia, 115 mg/dL; creatinine, 1.4 mg/dL; total cholesterol, 295 mg/dL; high-density lipoprotein (HDL-cholesterol), 56 mg/dL; low-density lipoprotein (LDL-cholesterol), 183 mg/dL; and triglycerides, 179 mg/dL.

**Figure 1:**
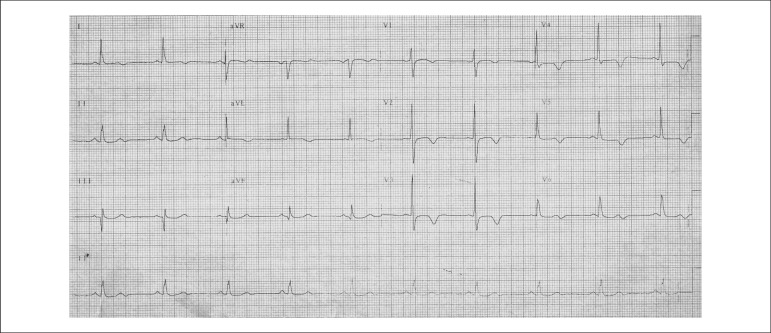
ECG. Sinus rhythm, probable electrically inactive inferodorsal area and diffuse ventricular repolarization changes.

Her coronary angiography (December 12, 1996) showed obstructions of 90% in the right coronary and circumflex arteries, and of 70% in the anterior interventricular artery, in addition to normal left ventricular motility.

On January 20, 1997, the patient was submitted to successful coronary angioplasty with stent implantation in the circumflex artery, and unsuccessful coronary angioplasty of the right coronary artery, which was occluded. On January 29, 1997, she underwent coronary angioplasty with stent implantation in the anterior interventricular coronary branch.

The patient became asymptomatic. On August 8, 1997, control coronary angiography evidenced occlusion of the right coronary artery, irregularities in the interventricular and circumflex arteries, and a 70% obstruction in the first branch of the left marginal artery.

The laboratory assessment (March 25, 1998) showed: triglycerides, 338 mg/dL; total cholesterol, 294 mg/dL; LDL-cholesterol, 181 mg/dL; and HDL-cholesterol, 45 mg/dL. On January 27, 2000, a new laboratory assessment evidenced: triglycerides, 166 mg/dL; total cholesterol, 289 mg/dL; LDL-cholesterol, 210 mg/dL; and HDL-cholesterol, 46 mg/dL.

Simvastatin was added to the ongoing fenofibrate.

The laboratory assessment (December 14, 2001) revealed: triglycerides, 239 mg/dL; total cholesterol, 268 mg/dL; LDL-cholesterol, 160 mg/dL; and HDL-cholesterol, 60 mg/dL.

The new coronary angiography (December 18, 2001) showed a 90% obstruction in the right coronary artery, and complicated with injury and thrombosis of the right brachial artery. Thromboembolectomy and brachial-brachial graft with the ipsilateral basilic vein were performed.

The patient remained asymptomatic until 2011, with the diagnosis of diabetes mellitus since 2008, when metformin and glybenclamide were prescribed.

On August 2011, the patient sought medical care complaining of angina on moderate exertion, being then submitted to coronary angiography (August 22, 2011), which showed: in-stent restenosis of 90% associated with an 80% obstruction in the mid third of the anterior interventricular artery; an 80% obstruction in the first branch of the diagonal artery; a 90% distal obstruction of the circumflex artery with patent coronary stent; an obstruction in the ostium of the second branch of the left marginal artery; a 90% obstruction of the right coronary artery; and preserved left ventricular motility.

On September 15, 2011, the patient had prolonged chest pain at rest, and her laboratory assessment showed: hemoglobin, 12.1 g/dL; red blood cell count, 37%; platelets, 348,000/mm^3^; creatinine, 1.11 mg/dL; potassium, 4 mEq/L; sodium, 140 mEq/L; triglycerides, 223 mg/dL; total cholesterol, 175 mg/dL; HDL-cholesterol, 47 mg/dL; LDL-cholesterol, 83 mg/dL; creatine kinase MB mass, 0.2 ng/mL; troponin, 0.064 ng/mL; activated prothrombin time (APT) according to International Normalized Ratio (INR), 1; and activated partial thromboplastin time ratio (APTT), 0.84.

The electrocardiography performed on September 15, 2011, revealed sinus rhythm, diffuse ventricular repolarization changes, and no changes of the special leads V_3r_, V_4r_, V_7_ and V_8_ (Figures 2 and 3).

**Figure 2:**
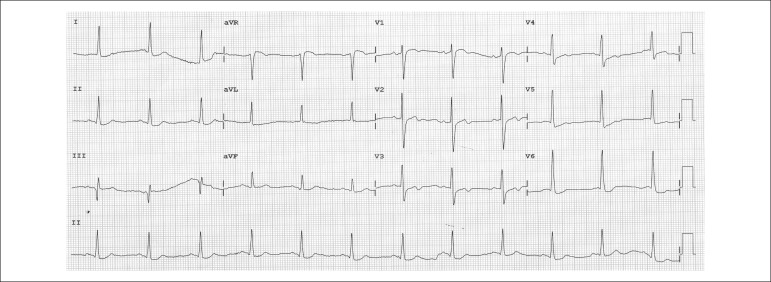
ECG. Sinus rhythm, diffuse ventricular repolarization changes.

**Figure 3:**
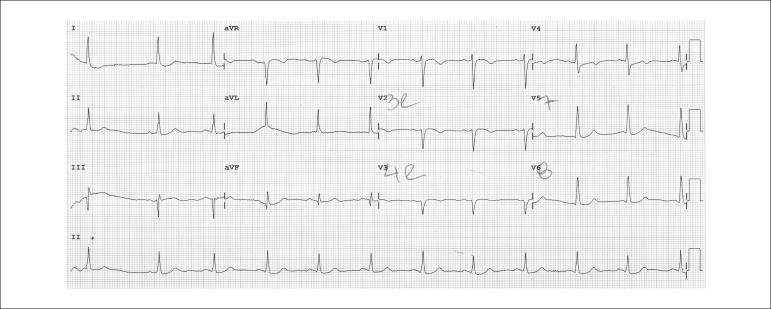
ECG. Right and dorsal leads, no evidence of ST-segment changes.

The sequential laboratory assessment on September 16, 2011, showed creatine kinase MB mass of 0.18 ng/mL and troponin of 0.34 ng/mL. On that same day, the patient underwent balloon angioplasty of the first diagonal branch, with stenting of the anterior interventricular artery. Bleeding, hemoglobin drop to 8.8 g/dL and pseudoaneurysm formation at the left femoral artery puncture site occurred.

On September 20 and 23, 2011, the pseudoaneurysm was injected with prothrombin, with resolution of local bleeding. The patient was discharged on September 26, 2011.

Two days after hospital discharge, she patient sought the emergency unit because of pain and left upper limb swelling.

The laboratory tests on September 28, 2011, showed: hemoglobin, 10.4 g/dL; red blood cell count, 32%; medium corpuscular volume (MCV), 103 fl; leukocytes, 102,310/mm^3^ (band neutrophils 36%, segmented neutrophils 62%, lymphocytes 1%, monocytes 1%); platelets, 292,000/mm^3^; creatine kinase MB mass, 0.27 ng/mL; troponin I, 0.126 ng/mL; creatinine, 2.52 mg/dL (glomerular filtration, 19 mL/min/1.73 m^2^); aspartate aminotransferase (AST), 77 U/L; alanine aminotransferase (ALT), 75 U/L; gamma glutamyl transferase, 179 U/L; total bilirubin, 1.05 mg/dL; direct bilirubin, 0.55 mg/dL; sodium, 136 mEq/L; potassium, 4.6 mEq/L; APT (INR), 1.3; and APTT, 1.04.

Doppler ultrasound showed venous thromboses in the left upper limb, and anticoagulant therapy was initiated.

A few hours later, the patient had atrial fibrillation with rapid ventricular response. After amiodarone infusion, she had dyspnea, consciousness lowering, respiratory failure requiring orotracheal intubation for ventilatory support, and cardiac arrest with pulseless electrical activity. She recovered, but showed high-grade atrioventricular block, atrial fibrillation, severe bradycardia and right QRS axis deviation.

On September 29, 2011, the laboratory tests were as follows: hemoglobin, 10.5 g/dL; red blood cell count, 35%; MCV, 109 fl; leukocytes, 9,820/mm^3^ (metamyelocytes 1%, band neutrophils 40%, segmented neutrophils 41%, eosinophils 1%, lymphocytes 14%, monocytes 3%); platelets, 202,000/mm^3^; creatine kinase MB mass, 13.19 ng/mL; troponin I, 1.22 ng/mL; urea, 108 mg/dL; creatinine, 2.82 mg/dL (glomerular filtration, 12 mL/min/1.73m^2^); sodium, 135 mEq/L; potassium, 5 mEq/L; AST, 756 U/L; ALT, 312 U/L; gamma glutamyl transferase, 136 U/L; total bilirubin, 1.19 mg/dL; direct bilirubin, 0.71 mg/dL; C-reactive protein, 209 mg/L; venous lactate, 105 mg/dL; APT (INR), 3.9; and APTT, 7.97. Venous blood gas analysis showed: pH, 6.55; pCO_2_, 33.9 mm Hg; pO_2_, 27.1 mm Hg; O_2 _saturation, 21.4%; bicarbonate, 2.8 mEq/L; and base excess, (-) 29.8 mEq/L.

The patient underwent temporary pacemaker implantation, but the refractory shock persisted. She had a new cardiac arrest and no longer responded to resuscitation maneuvers, dying on September 29, 2011.

## Clinical aspects

The patient is an 88-year-old female, who, because of chest angina, underwent percutaneous angioplasty with stenting, remaining asymptomatic for 13 years. The angina reappeared, and, after a new percutaneous intervention, she died. She underwent seven coronary angiographies, three of which with angioplasty and stenting. The fifth and seventh procedures had complications.

The first point of interest is the indication of intervention therapy with stenting to an elderly patient with chest angina. Published studies support that indication. The results of intervention therapy in patients older than 80 years (n = 983) have shown a four-year survival of 71.6% for stent implantation (n = 289), better than that achieved with isolated drug treatment (n = 561, survival of 60.3%)^1^. The good results of the intervention therapy were later confirmed in 79/276 patients (52%) aged 80 ± 4 years, whose four-year survival was 72%^2^. The clinical experience confirms those observations. Thus, in our patient, the percutaneous intervention therapy performed for the first time at the age of 74 years and followed by 13 asymptomatic years was again indicated, when she became symptomatic.

The patient underwent seven coronary angiographies in 15 years of follow-up, three of which (42.9%) with percutaneous coronary intervention via brachial and femoral punctures. Two of those revascularizations (66.7%) complicated with thrombosis, bleeding and pseudoaneurysm formation, requiring surgery for thromboembolectomy in the first event, and thrombin administration in the pseudoaneurysm and expectant management in the second event.

The second point of interest is performing coronary angiography in octogenarians, whose mortality is higher (0.8%) than that of the general elderly population (0.11%). In that age group, the risk of vascular complications, such as arterial occlusions requiring surgical correction or thrombectomy, retroperitoneal bleeding, formation of hematoma, pseudoaneurysm and arteriovenous fistula, as well as the risk of infection, is greater (5%). Studies have confirmed that the diagnosis of femoral pseudoaneurysm occurred in more than 0.2% of the cases, in 8% of the catheterization processes^3^, the risk of pseudoaneurysm formation being higher in cases whose compression of the femoral artery access was performed with a device (39 in 1768 events – 2.2%) as compared to those with manual compression (1720 events/1.7%) at the puncture site^4^. A meta-analysis has confirmed the risk of bleeding at the femoral artery puncture site, comparing compression with devices (n = 1700) to manual compression (n = 1500), the risks being 4.6% and 4.1%, respectively. The estimated risk of an intervention at the puncture site is 1.6-fold higher with compression with devices than with manual compression. The risks of blood transfusion and of arterial ischemia in the lower limb for compression with devices were, respectively, 1.2 and 2.1 times greater than those using manual compression. Despite the nonsignificant results, complications were more frequently found when using compression with devices (3.8%) at the arterial puncture site than when using manual compression (1.7%)^4^.

In the case discussed, the patient being of the female sex and submitted to therapeutic percutaneous coronary intervention increased the risk for pseudoaneurysm formation^5^. The expectant management of the femoral pseudoaneurysm assumes a vascular diameter smaller than 2 cm, and the reason for not using ultrasound-guided percutaneous injection of thrombin might have been the possibility of spontaneous thrombosis of the vascular content^5^.

The third point of interest is the treatment of elderly patients with coronary artery disease and its impact on survival. A study of 7472 octogenarians (mean age of 83 years) undergoing percutaneous coronary intervention has reported mortality ranging from 0 to 19%, being around 5% for patients older than 85 years, the mortality predictors being as follows: cardiogenic shock (31%); acute myocardial infarction (11%); lower ejection fraction (35%); kidney failure (7.2%); first coronary intervention (2.7%); patients older 85 years (1.8%); and diabetes mellitus (1.2%)^6^.

The recommendation of optimized drug treatment as the initial option for patients with chronic coronary artery disease is supported by a meta-analysis with 63 studies (1852 symptomatic and asymptomatic patients diagnosed with chronic coronary artery disease, mean age ranging from 56 to 65 years), comparing four possibilities of procedures (percutaneous coronary intervention *versus* drug treatment; angioplasty with stent *versus* conventional balloon angioplasty; angioplasty with stent *versus* drug treatment; angioplasty with drug eluting stent *versus* angioplasty with bare-metal stent), with no difference in mortality, acute myocardial infarction, coronary artery bypass graft surgery or need for a new procedure within 12 months^7^.

In the case discussed, 15 years after coronary disease stratification and percutaneous coronary intervention, the patient had angina on moderate exertion, suggesting that the therapeutic strategy with percutaneous and drug revascularization, to control risk factors, did not prevent disease progression.

Considering that the patient had coronary artery disease and diabetes mellitus, the clinical experience confirms the greater risk for restenosis and occlusion after revascularization procedures, via either percutaneous coronary intervention or coronary artery bypass graft surgery, as compared to nondiabetic patients with multivessel coronary disease. Hlatky et al.^8^ have reported similar mortality for patients undergoing coronary artery bypass graft surgery (575/3889 patients) and for those (628/3923) undergoing percutaneous coronary intervention, 15% and 16%, respectively. In patients older than 65 years, however, treatment changed mortality. In patients with diabetes, the mortality of those undergoing coronary artery bypass graft surgery (615 patients) was substantially lower than that of those (618 patients) undergoing percutaneous coronary intervention, suggesting that coronary artery bypass graft surgery yields lower mortality in patients with diabetes older than 65 years^8^.

The controversy over the therapeutic option for the elderly is evident when assessing the result of two studies performed in patients older than 75 years with coronary artery disease. The Italian Elderly Acute Coronary Syndrome Trial Investigators has compared the survival of patients treated with an early invasive approach *versus* the clinical conservative approach, evidencing no advantage of the initial aggressive therapy^9^. Another recent study with patients diagnosed with non-ST-elevation acute coronary syndrome has estimated the presence of events (mortality, myocardial infarction, stroke, re-hospitalization due to cardiovascular cause, or bleeding) in the invasive treatment (86/182 patients; 47.3% females) as compared with that in the conservative approach (70/131 patients; 53.4% females). That study has shown those events in 24.7% of the patients undergoing early invasive therapy (45/182 patients) as compared to 40.5% (53/131 patients) of those undergoing the initial conservative treatment in a one-year follow-up. The patients undergoing invasive treatment had an improvement in survival with a reduction in death/nonfatal infarction (14.3% or 26 patients) and in new hospitalizations (9.9% or 18 patients) as compared to those undergoing conservative treatment (27.5% or 36 patients, and 16.8% or 22 patients, respectively)^10^.

It is worth noting that, although stents efficiently reestablish the vascular lumen by reducing 50% of the angiographic restenosis, they cause injure to the vascular wall, and, via repairing mechanisms, a healing response that, depending on the severity of the process, will lead to reobstruction of the vessel treated. The analysis of the composition of 40 coronary thrombi manually aspirated in the first 4 to 16.5 hours from chest pain onset, during primary percutaneous coronary intervention, has identified the presence of fibrin (49.1%), red blood cells (24.2%), platelets (11.6%) and leukocytes (3.7%) in the material studied^11^.

According to Montalescot et al., although current interventional treatments reduce the risk of restenosis by as much as 40% as compared to previous techniques, the introduction of conventional stent has not improved survival as compared to balloon angioplasty^12^. In addition, the use of drug-eluting stents has not improved survival as compared to conventional stents^13^.

The patient underwent new percutaneous coronary intervention with coronary stenting in the anterior interventricular artery and balloon angioplasty in the first branch of the diagonal artery, and had immediate and late, local and systemic complications of the procedure (bleeding at the puncture site with pseudoaneurysm formation in the left femoral artery and venous thrombosis in the left upper limb).

The acute drop in hemoglobin after the percutaneous intervention (the seventh procedure), as well as stent implantation with evidence of macrocytosis and elevation of the MCV can be associated with excessive regeneration of bone marrow or with altered DNA synthesis, probably related to post-hemorrhage anemia. The presence of leukocytosis with 36% of band neutrophils can be explained as stimulation by the inflammatory process, trauma or necrosis with release of interleukin-1, mobilization of the bone marrow reserve pool of band neutrophils and “shift to the left”. The absence of neutrophilia can be explained by the patient’s age group, suggesting the possibility of an infectious process.

Analyzing the laboratory tests, the increase in serum creatinine with a reduction in glomerular filtration (19 mL/min/1.73 m^2^) and rapid progression with elevation in aminotransferases suggest renal injury with severe renal failure and acute liver failure due to ischemia/hypoxia and prolonged hypotension.

It is worth noting that, although the two sequential measurements of troponin (0.126 ng/mL and 1.22 ng/mL) performed at a 24-hour interval were within the normal range, a significant increase in the second value was observed, which can suggest an increased cardiovascular risk. That risk was confirmed in a study with 2285 patients diagnosed with stable coronary artery disease and diabetes mellitus (female sex, 34.9%; age range, 55 to 68 years; mean age, 61 years), and followed up for five years^14^. Cardiovascular death, non-fatal acute myocardial infarction or non-fatal stroke occurred in 12.9% (178/1388 patients) of those with normal troponin as compared to 27.1% (243/897 patients) of those with elevated troponin. An elevation in troponin concentration greater than 25% in a four-year follow-up proved to be an independent marker of cardiovascular risk^14^.

The patient had atrial fibrillation with rapid ventricular response and developed hemodynamic instability, respiratory instability and cardiac arrest after amiodarone intravenous infusion. Although the clinical experience confirms the efficacy of amiodarone to reverse atrial fibrillation in 80% of the cases, it should be carefully used in elderly patients, with special attention paid to its infusion velocity. Two authors have assessed the incidence of atrial fibrillation and the survival of patients with that arrhythmia. Reinel et al. have studied 310 episodes of arrhythmia in 133 severely ill patients, 29.8% of which (83/278 episodes of tachycardia) represented atrial fibrillation. Baine et al. have studied the incidence of types of arrhythmia in patients (144512) older than 65 years, atrial fibrillation being the most frequently found cardiac rhythm (44.8%)^15^. In another study of 4060 patients with atrial fibrillation and mean age of 69.7 years (range, 60.7 – 78.7), the mortality of those with controlled cardiac rhythm was 17.5% (356/2033 patients) as compared to 15.3% (310/2027 patients) among those with controlled heart rate^16^.

The fourth point of interest was the patient’s sudden clinical deterioration, suggesting the release of mediators, causing hypoxemia and hypotension requiring the differential diagnosis between sepsis and pulmonary thromboembolism. The rapid course with cardiac arrhythmia, hypotension, severe hypoxemia and cardiac arrest with pulseless electrical activity can be associated with massive pulmonary embolism^17^. A study has confirmed that 79% of the patients with pulmonary embolism had evidence of deep venous thrombosis of the lower limbs, and, of the hereditary or acquired risk factors, advanced age and antiphospholipid antibody syndrome increased the likelihood of acute episodes of repeated deep venous thrombosis and pulmonary embolism^18^.

The cause of death was pulmonary thromboembolism. The patient with chronic coronary artery disease and several cardiovascular risk factors, such as arterial hypertension, hypercholesterolemia and diabetes mellitus, showed progressive coronary atherogenesis. In one decade, she had repeated episodes of vascular thrombosis, the last thromboembolic event occurring at an uncommon site (upper limb), suggesting the need for thrombophilia assessment. The patient had cardiac arrhythmia, hemodynamic instability and refractory cardiogenic shock probably due to massive pulmonary embolism, and died. **(Magaly Marçula, MD)**

**Diagnostic hypotheses:** pulmonary thromboembolism, deep venous thrombosis at an uncommon site, chronic coronary artery disease. Cause of death: cardiogenic shock. **(Magaly Marçula, MD)**

## Necropsy

**Figure 4:**
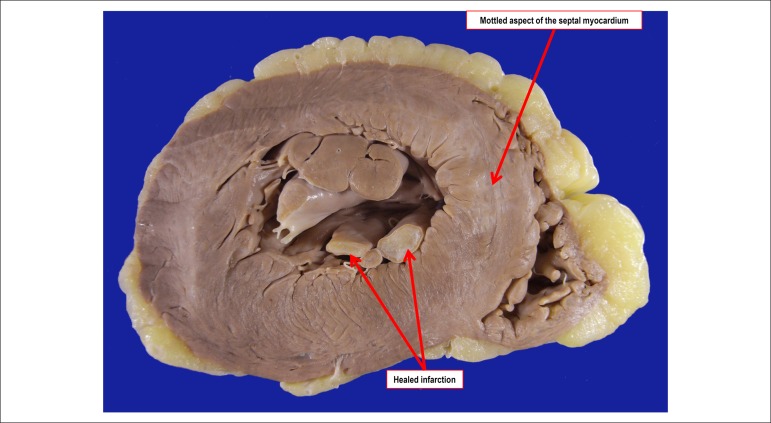
Cross section of the ventricles showing mild left ventricular hypertrophy, in addition to irregularly mottled septal area and whitish area in the posteromedial papillary muscle (old healed infarction).

**Figure 5:**
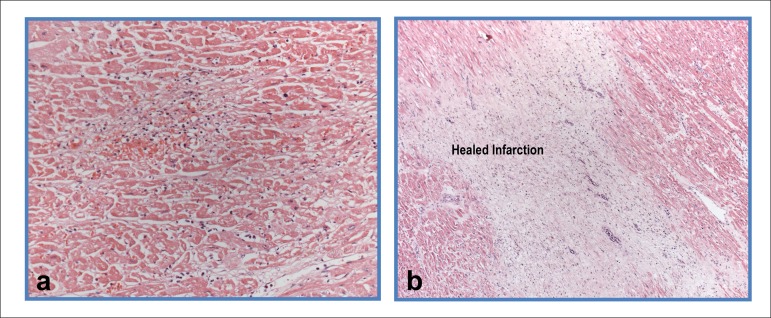
Photomicrographs of the ventricular myocardium showing: a) area of organizing infarction corresponding to the mottled ventricular septum area; b) fibrotic and scarring area in the inferior wall. Hematoxylin-Eosin, 20X and 5X, respectively.

**Figure 6:**
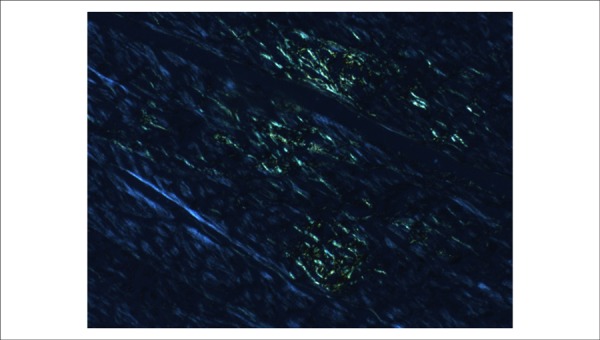
Polarized light photomicrographs of the ventricular myocardium. Greenish areas correspond to amyloid substance deposits. Congo red under polarized light, 10X.

**Figure 7:**
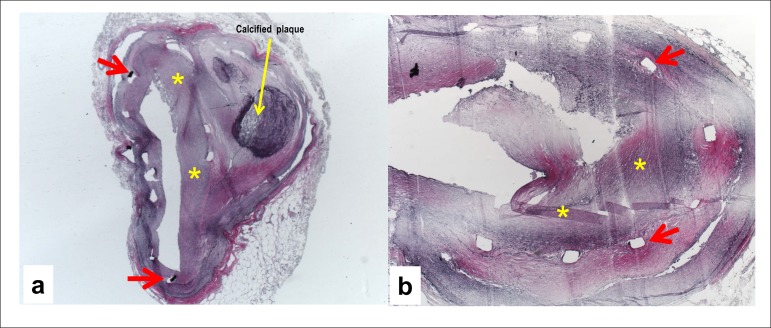
Photomicrographs of segments of the left coronary artery anterior interventricular branch, where signs of the old stent rods are seen (rectangular regions, usually empty due to artifactual detachment of the stent during the section, but sometimes partially filled with a dark residual material from the stent). There is fibrous neointimal thickening inside the old stent (neoatherosclerosis with fibrous plaque, marked with asterisks). In addition, residues of the older calcified plaque, external to the stent (yellow arrow in panel a) can be seen. Verhoeff stain for elastic fibers, 2.5X and 5X, respectively.

**Figure 8:**
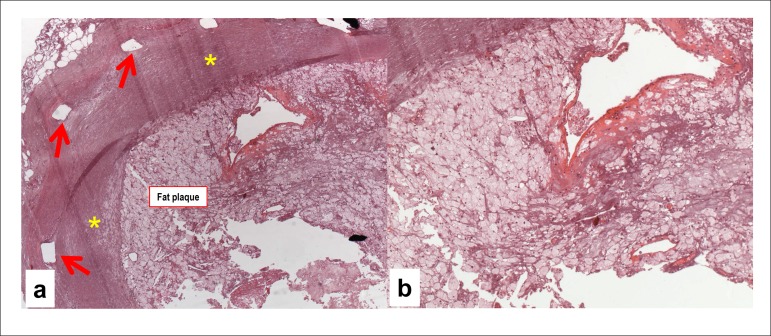
Photomicrographs of segments of the left coronary artery circumflex branch, where signs of the old stent rods are seen (rectangular regions, usually empty due to artifactual detachment of the stent during the section). Note, inside the stent (panel a), neoatherosclerosis with mixed plaque, fibrous (asterisks) and fatty, rich in xanthomatous histiocytes (detail in panel b). Hematoxylin-Eosin, 5X and 10X, respectively.

The heart weighed 453 g (normal for the female sex: up to 350 g). Its opening revealed hypertrophy of the left ventricular walls and mottled aspect of the ventricular septum myocardium (Figure 4). On microscopic examination, the mottled area corresponded to 2-to-3-week-old myocardial infarction (Figure 5). Fibrotic or scarring foci and focal areas of extracellular amorphous substance accumulation with staining characteristics of amyloid (Congo red) were identified (Figure 6). The microscopic study of the coronary arteries revealed recent partial thrombosis on the seventh cm of the right coronary artery, in addition to atherosclerosis with 80% obstruction. The anterior interventricular and circumflex branches of the left coronary artery had stents placed several years before and were submitted to special processing with resin inclusion, allowing for histological sections. These sections showed fibrous (anterior interventricular branch) or fatty (circumflex branch) atherosclerotic plaques in the coronary arteries causing in-stent occlusion (Figures 7 and 8).

**Figure 9:**
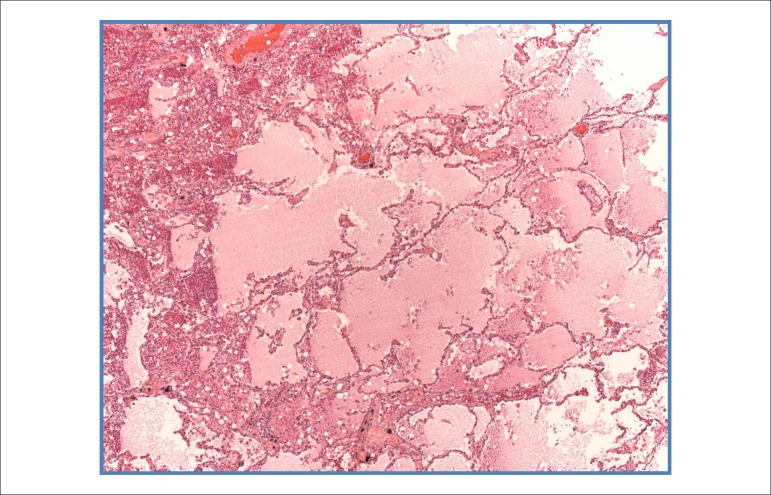
Photomicrographs of intra-alveolar edema in the lungs. Hematoxylin-Eosin, 5X.

The lungs weighed together 860g, and their microscopic study showed areas of alveolar edema (Figure 9). In addition, thromboemboli were identified in small intraparenchymal arteries with Gram-positive cocci. There was terminal bronchopneumonia, not related to the septic thromboembolism.

**Anatomopathological diagnoses:** moderate systemic and coronary atherosclerosis with in-stent atheroma plaques; cardiovascular amyloidosis; pulmonary alveolar edema; terminal bronchopneumonia; and foci of septic pulmonary thromboembolism.

**Cause of death:** organizing myocardial infarction in the ventricular septum **(Vera Demarchi Aiello, Prof. MD)**

## Comments

Coronary arteries submitted to stent implantation can develop late in-stent restenosis, due to neoatherosclerosis, which happened in the case reported. The resin inclusion technique allowed for sectioning and assessing the stented coronary segments. There were atherosclerotic plaques of varied composition (fibrous and fatty). That obstruction accounted for the recent septal infarction. Despite the occlusive lesions in the right coronary artery, which was not the dominant vessel in this case, no infarction was identified in that coronary artery territory.

A very recent study by Taniwaki et al.^19^ has shown that in-stent neoatherosclerosis was more frequently found in patients with clinical and angiographic evidence of atherosclerosis progression in nontreated native coronary segments. Cardiovascular amyloidosis was an occasional finding, and, although not extensive, it might have contributed to the final myocardial dysfunction.

The lungs showed focal alveolar edema and terminal bronchopneumonia. The source of the septic thromboemboli was not identified. The gross examination of the heart evidenced no signs of infectious endocarditis that could explain that embolic event. According to clinical data, there were clinical signs of venous thrombosis of the left upper limb, which, if infected, could have been the embolic source. Usually the exposed areas are not inspected on the postmortem examination, explaining why that thrombosis was not studied. **(Vera Demarchi Aiello, Prof. MD)**

**Editor da Seção:** Alfredo José Mansur (ajmansur@incor.usp.br)

**Editores Associados:** Desidério Favarato (dclfavarato@incor.usp.br)

Vera Demarchi Aiello (anpvera@incor.usp.br)
